# Degradation of gaze‐speech synchronization due to mild cognitive impairment in picture description tasks

**DOI:** 10.1002/alz.092533

**Published:** 2025-01-03

**Authors:** Michael J. Kleiman, James E Galvin

**Affiliations:** ^1^ University of Miami Miller School of Medicine, Boca Raton, FL USA

## Abstract

**Background:**

When performing a picture description task, healthy individuals tend to look only briefly at a target before beginning its description, after which they move promptly onto the next target. This sequence may be disrupted in those with cognitive impairment. Just as cognitively impaired individuals produce greater numbers of disfluencies and pauses, those with mild cognitive impairment (MCI) may delay speech production by extending their gaze behavior towards a target before beginning its description.

**Method:**

To explore this, we examined the gaze and speech behavior of 39 participants (27 HC, 12 MCI) while engaging in the Cookie Theft picture description task. Transcriptions with word‐level timings were generated using OpenAI’s Whisper in combination with the *whisper‐timestamped* Python package utilizing Dynamic Time‐Warping for more accurate timestamps with reduced hallucinations. Gaze was collected using a Tobii Pro Spectrum 300Hz eye tracker and processed using custom‐developed fixation filters, with gaze target timing identified as the first full fixation directed towards that area of interest. Speech timing was determined as the beginning of the sentence containing the target description.

**Result:**

MCI participants initiated speech production after a longer delay following target fixation than HC participants, and produced shorter and less descriptive responses. Gaze behavior was also more sluggish in MCI participants; visits onto targets were less frequent and longer, and saccadic movements between targets were less frequent.

**Conclusion:**

The intersection of gaze and speech behavior is a potentially powerful avenue for identifying early‐stage cognitive impairment, but is currently under‐utilized. Future research in gaze‐speech synchronization may identify patterns that could target biomarker‐confirmed preclinical Alzheimer’s disease.

